# Supplementary material for: Contribution of Whole-Genome Sequencing and Transcript Analysis to Decipher Retinal Diseases Associated with MFSD8 Variants
Source: Int J Mol Sci. 2022 Apr 13;23(8):4294. doi: 10.3390/ijms23084294 (PMC9032189; doi:10.3390/ijms23084294)
Supplement: Supplementary file 1 [file ijms-23-04294-s001.zip › Poncet et al_Supplementary Figures Legends_revised.pdf]

Contribution of whole genome sequencing and transcript analysis to decipher retinal diseases associated with *MFSD8* variants.

Supplementary Figures Legends

Supplementary Figure S1: Full-field ERG of patients with isolated retinal degeneration linked to *MFSD8*. Top, Patient L-08031428 had a reduction of dark-adapted (DA 0.01, DA 3.0 and DA 10.0) responses and a more marked reduction of light adapted (LA 3.0 and LA 3.0 Flicker) responses. DA 3.0 and DA 10.0 responses depicted supplementary reduction of b/a ratio. Cone-rod dystrophy. At follow-up, supplementary reduction of both dark- and light-adapted responses. MonPackOne, Métrovision, Pérenchies, France. Bottom, patient VV-51717 had a normal ffERG at initial evaluation.

Supplementary Figure S2: Molecular diagnosis flowchart in the Lille cohort. The 3,348 probands of the Lille cohort underwent a genetic analysis, either by next generation sequencing (NGS) of a large gene panel including *MFSD8* gene (n=1,049), or by NGS of a small panel of targeted genes not including *MFSD8* (n=2,299). Each category is divided into two groups: a group named "other IRDs" with patients presenting Rod-Cone Dystrophy, Leber Congenital Amaurosis, Congenital Stationary Night Blindness, Choroideremia (n= 709 analyzed by a large NGS panel; n= 1,346 analyzed by a small NGS panel); and a second group with patients presenting Cone Dystrophy, Macular Dystrophy including Stargardt disease (n= 340 analyzed by a large NGS panel; n= 953 analyzed by a small NGS panel. *ABCA4* is the most frequent gene identified in this group with 549 STGD1 cases identified by the small panel and 48 by the large panel (n=597). The presence of other patients harbouring *MFSD8*

variants among the 356 unsolved patients with CD or MD, analyzed by a small panel cannot be ruled out. The two swiss patients described in this study were not part of this initial cohort.

Supplementary Figure S3: Novel *MFSD8* variants identified by whole genome sequencing.

a) The deep intronic variant c.998+1669A>G was found in intron 10 of *MFSD8* in patient HD-OPH1206. **b)** Patient L-20021807 was heterozygous for a large deletion encompassing exons 9 and 10 of the gene.

Supplementary Figure S4: Impact of variants on MFSD8/CLN7 3D structure based on Alphafold website: https://alphafold.ebi.ac.uk/entry/Q8NHS3. The localization of all missense variants identified in the study is presented. Two sides of the protein are depicted in **a)** and **b)**
