# Supplementary material for: Contribution of Whole-Genome Sequencing and Transcript Analysis to Decipher Retinal Diseases Associated with MFSD8 Variants
Source: Int J Mol Sci. 2022 Apr 13;23(8):4294. doi: 10.3390/ijms23084294 (PMC9032189; doi:10.3390/ijms23084294)
Supplement: Supplementary file 1 [file ijms-23-04294-s001.zip › Supplementary Figure S2.pdf]

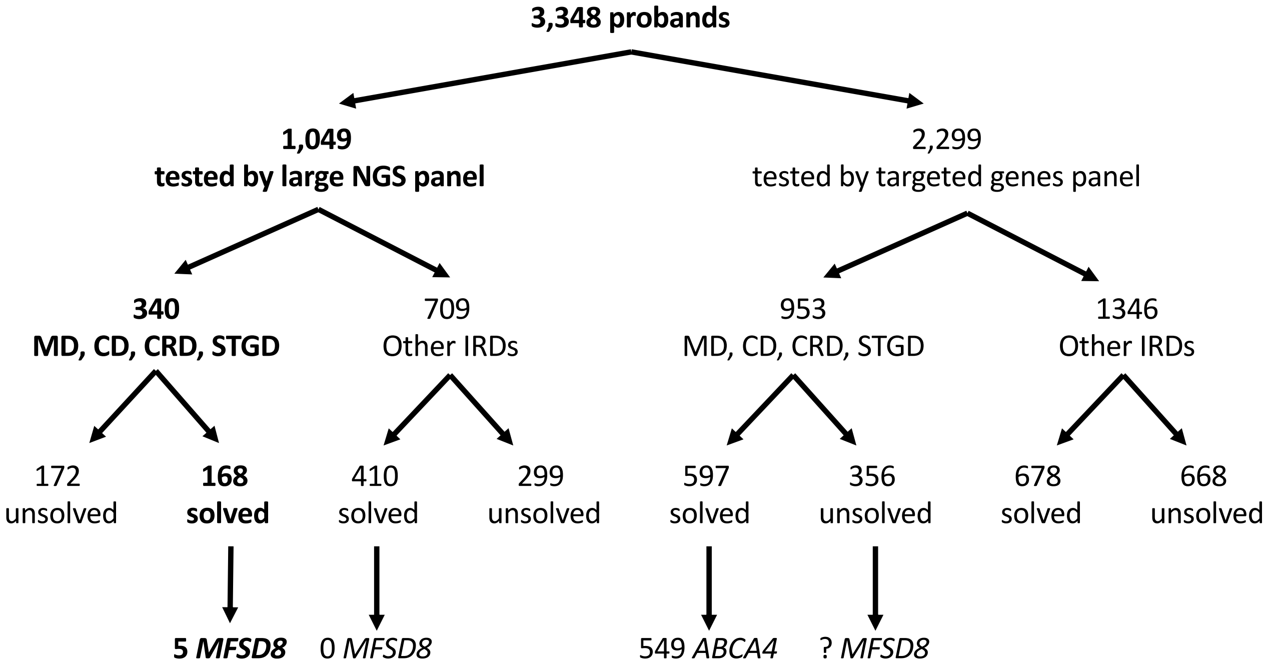

3,348 probands
1,049
tested by large NGS panel
2,299
tested by targeted genes panel
340
MD, CD, CRD, STGD
709
Other IRDs
953
MD, CD, CRD, STGD
1346
Other IRDs
172
unsolved
168
solved
410
solved
299
unsolved
597
solved
356
unsolved
678
solved
668
unsolved
5 MFSD8
0 MFSD8
549 ABCA4
? MFSD8

Figure S2: The 3,348 probands of the Lille cohort underwent a genetic analysis, either by next generation sequencing (NGS) of a large gene panel including *MFSD8* gene (n=1,049), or by NGS of a small panel of targeted genes not including *MFSD8* (n=2,299). Each category is divided into two groups: a group named "other IRDs" with patients presenting Rod-Cone Dystrophy, Leber Congenital Amaurosis, Congenital Stationary Night Blindness, Choroideremia (n= 709 analyzed by a large NGS panel; n= 1,346 analyzed by a small NGS panel); and a second group with patients presenting Cone Dystrophy, Macular Dystrophy including Stargardt disease (n= 340 analyzed by a large NGS panel; n= 953 analyzed by a small NGS panel. *ABCA4* is the most frequent gene identified in this group with 549 STGD1 cases identified by the small panel and 48 by the large panel (n=597). The presence of other patients harbouring *MFSD8* variants among the 356 unsolved patients with CD or MD, analyzed by a small panel cannot be ruled out. The two swiss patients described in this study were not part of this initial cohort.
