# Supplementary material for: Contribution of Whole-Genome Sequencing and Transcript Analysis to Decipher Retinal Diseases Associated with MFSD8 Variants
Source: Int J Mol Sci. 2022 Apr 13;23(8):4294. doi: 10.3390/ijms23084294 (PMC9032189; doi:10.3390/ijms23084294)
Supplement: Supplementary file 1 [file ijms-23-04294-s001.zip › Supplementary Table S1.pdf]

Supplementary Table S1: Progression of retinal degeneration.

Patient, sex	Age at last examination	BCVA at last examination	Fundus	Visual Field	SW-FAF	SD-OCT	Other
L-08031428, M	28	OU: 20/800	Waxy pallor of ONH<br>Vascular narrowing<br>Bull's eye macular lesion<br>Whitish discoloration of midperipheral retina<br>Bone spicules in nasal midperipheral retina	40° large absolute (V4e) central scotoma; preservation of peripheral V4e isopter	Large macular hypoAF round lesion with indistinct hyperAF edges<br>Large midperipheral ring of hypoAF with indistinct hyperAF edges	Widespread disappearance of outer reflective layers (ONL, EZ, RPE)<br>Dense irregular deposits at the level of RPE	ffERG: residual dark-adapted responses; unrecordable light-adapter responses
VV-51717, M	57	VA: 0.2 RE, 0.1 LE	Bull's eye maculopathy	Not repeated	Macula: hypoAF area, surrounded by hyperAF ring<br>No changes in peripheral retina	Disappearance of outer layers (ONL, EZ, IS/OS, RPE) in the macula<br>Dense irregular deposits at the level of RPE	
VV-1595021, F	20	VA: 0.2 RE, 0.25 LE	Central foveal atrophy, peripheral retina within normal limits	Not repeated	Macula: hypoAF area, surrounded by hyperAF ring<br>No changes in peripheral retina	Disappearance of outer layers (ONL, EZ, IS/OS RPE) in the macula<br>Dense irregular deposits at the level of RPE	
HD-OPH4200, F	53	VA :<br>RE : 0.2, P8<br>LE : 0.2, P8<br>Eccentric viewing	Loss of foveal reflex with a few discreet hypo-pigmented spots around the fovea	Bilateral relative central scotoma	Macula: hypoAF area, surrounded by hyperAF ring<br>No changes in peripheral retina	Disappearance of outer layers (ONL, EZ, IS/OS, RPE) in the macula	Major photophobia
HD-OPH1206, M	36	VA :<br>RE : 0.3ff ; P4f<br>LE : 0.3 f ; P5	Bull's eye maculopathy	Bilateral relative central scotoma	Macula: hypoAF area, surrounded by hyperAF ring<br>No changes in peripheral retina	Disappearance of outer layers (ONL, EZ, IS/OS, RPE) in the macula<br>Dense irregular deposits at the level of RPE	
