# Supplementary material for: Contribution of Whole-Genome Sequencing and Transcript Analysis to Decipher Retinal Diseases Associated with MFSD8 Variants
Source: Int J Mol Sci. 2022 Apr 13;23(8):4294. doi: 10.3390/ijms23084294 (PMC9032189; doi:10.3390/ijms23084294)
Supplement: Supplementary file 1 [file ijms-23-04294-s001.zip › Supplementary Table S2.pdf]

Supplementary Table S2 : Primers used in this study

Name	5’ Sequence 3’	
MFSD8_F	CCTTTTTGAAACCATCATTACTC	Transcript analysis for P1 LCLs
MFSD8_R	TATTGTGCAAATCTTCCCACTG	
MFSD8_syn_F	TCTAGGTCCAGTTTTTCAGACTTG	Transcript analysis for P2 and P3 LCLs
MFSD8_syn_R	GGAGTAATGATGGTTTCAAAAAGG	
GAPDH_RT_F	ACCCAGAAGACTGTGGATGG	Transcript analysis for Housekeeping gene in LCLs
GAPDH_RT_R	TTCTAGACGGCAGGTCAGG	
