# Supplementary figures and images for: Contribution of Whole-Genome Sequencing and Transcript Analysis to Decipher Retinal Diseases Associated with MFSD8 Variants
Source: Int J Mol Sci. 2022 Apr 13;23(8):4294. doi: 10.3390/ijms23084294 (PMC9032189; doi:10.3390/ijms23084294)

**a)**

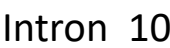

**b)**

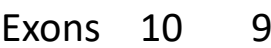

Supplement: Supplementary file 1 [file ijms-23-04294-s001.zip › Supplementary Figure S3.pdf]

Supplementary Figure S4

a)

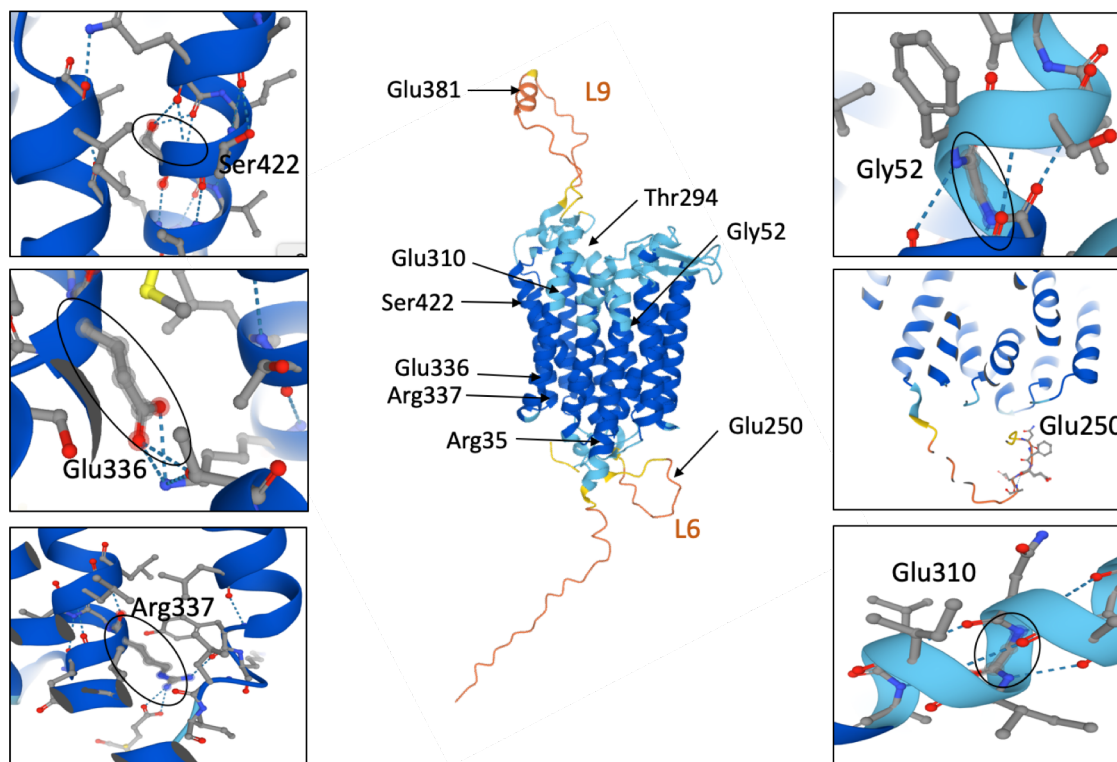

b)

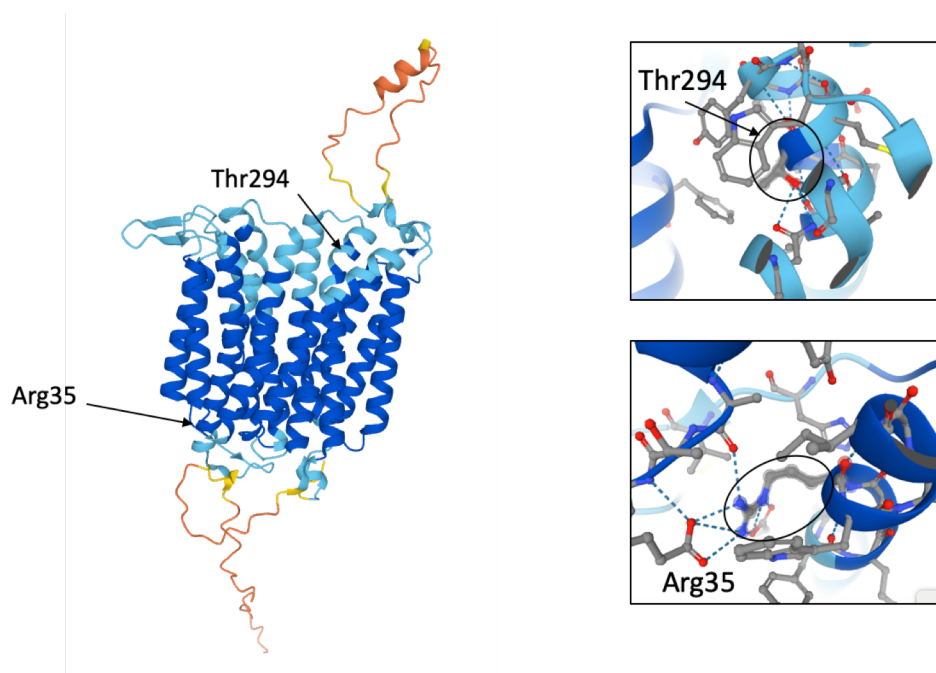

Supplement: Supplementary file 1 [file ijms-23-04294-s001.zip › Supplementary Figure S4.pdf]
